# Epigenetic remodeling: unveiling the potential endogenous mechanisms of exercise in alleviating neuropathic pain

**DOI:** 10.3389/fnins.2025.1622894

**Published:** 2025-09-05

**Authors:** Hao Shan, Yuntao Xu, Jiabao Guo, Tong Li

**Affiliations:** ^1^Department of Clinical Laboratory, Jiangsu Rongjun Hospital, Wuxi, Jiangsu, China; ^2^The Second School of Clinical Medical College, Xuzhou Medical University, Xuzhou, Jiangsu, China

**Keywords:** neuropathic pain, epigenetic, exercise, nerve injury, DNA methylation

## Abstract

Although exercise can improve neuropathic pain (NP), its underlying mechanisms have not been elucidated completely. Epigenetics involves the study of environmental factors, such as diet, temperature, and exercise, and basic functions. NP can be improved by controlling the state of epigenetic modification. This article focuses on the exercise and epigenetic mechanisms of NP and discusses the progress of the research on epigenetic regulation in the field of exercise rehabilitation. The studies presented herein are not only used to facilitate the understanding of the important potential mechanisms of exercise for improving NP but also provide a new idea that NP can be improved by endogenous regulatory mechanisms.

## 1 Introduction

In 2011, a group of professionals specializing in neuropathic pain (NP) in the International Association for the Study of Pain updated the definition of NP to pain caused by direct damage or illness in the somatosensory system (Haanpää et al., 2011). Approximately 7%–10% of people worldwide suffer from NP, and 17% of patients surveyed evaluated their quality of life as “more terrible than die” (Torrance et al., 2014; van Hecke et al., 2014). Methods for treating NP include drug therapy, minimally invasive interventional therapy, neuroregulatory therapy, and psychotherapy; however, in clinical studies, only a few patients can benefit from drug therapy (Hussain et al., 2020). The development of therapeutic strategies for NP is limited by complex pathophysiological mechanisms, and standards for diagnosis and prediction markers are lacking (Alsaloum et al., 2020; Finnerup et al., 2021). Although the molecular mechanism of NP has not been completely understood, evidence shows that exercise has massive potential to treat NP (Dhawan et al., 2020; Maharaj and Yakasai, 2018; Sayed and Abdel, 2014). Epigenetic modification builds a bridge between lifestyle factors, such as diet and exercise, and genes. Epigenetics can buffer the influence of lifestyle on the body and allow the body to exhibit a plastic response to lifestyle (Światowy et al., 2021). Exercise can affect NP by regulating and controlling gene expression (Cavalli and Heard, 2019). The present article reviews the role of epigenetic modification in NP and the epigenetic mechanism of exercise therapy for NP to provide its theoretical foundation.

## 2 Types of epigenetic modification and their roles in the nervous system

The developments and functions of the nervous system, such as how the brain develops and ages and how cognition is inherited across generations, can be elucidated within a new framework that is built by epigenetics. The occurrence of neurological diseases involves epigenetic mechanisms, including DNA methylation, non-coding RNA regulation, and histone and mRNA modification. Some environmental factors, such as diet, exercise, and life pressure, are the trigger mechanisms controlling gene expression under epigenetic modification and cause neurological diseases.

DNA methylation is a main mechanism in neurological diseases. It can regulate the expression patterns and stability of the genome, although DNA sequences themselves do not change. DNA methylation plays an important role in the development and maturation of the nervous system (Ladd-Acosta et al., 2007). Siegmund et al. (2007) evaluated the changes in 50 genetic loci associated with development, maturation, and senility in the temporal neocortex of the human brain in 125 volunteers aged 17–104 years. The DNA methylation of these loci is regulated by human activity and learning throughout the whole life cycle.

Non-coding RNAs, including microRNAs (miRNAs), long non-coding RNAs (lncRNAs), and circular RNAs (circRNAs), are RNA molecules that do not encode proteins. miRNAs are abundantly expressed in the central nervous system, where they play critical roles in regulating neuronal proliferation, differentiation, migration, and development. Furthermore, they are sensitive to environmental stimuli and can modulate signal transmission within neural networks by regulating the expression of downstream target genes (Thomas et al., 2018). lncRNAs regulate neuronal activities. Li P. et al. (2020) found that the upregulation of lncRNA H19 can augment C–C motif chemokine ligand 2 expression by sponging miR-1-3p and can then promote the proliferation and activation of astrocytes. circRNAs can control neuronal function by serving as miRNA sponges and through other mechanisms. Chen W. et al. (2020) discovered that circSHOC2 can act as an miR-7670-3p miRNA sponge, and the miR-7670-3p/SIRT1 axis can reduce neuronal apoptosis and damage. Reducing circHIPK2 could promote the differentiation of neural stem cells into neurons and enhance neuroplasticity (Wang et al., 2020).

Histone modification is a more dynamic posttranslation process than DNA methylation. It involves various chemical modifications, such as methylation, phosphorylation, acetylation, and ubiquitination, under external stimuli and alters the 3D structure of chromosomes, thereby affecting gene expression. Histone modification plays an important role in neuron development, plasticity, and behavioral memory (Cho and Cavalli, 2014; Geng et al., 2021).

mRNAs are another research hotspot in epigenetics. N6-methylation (m6A) is the most common mRNA modification (Boccaletto et al., 2022). It regulates mRNA cutting, transport, translation, degradation, and stability through specific recognition and combination with m6A recognition proteins at modification sites. Modification levels can be dynamically regulated by methyltransferases, such as methyltransferase-like protein 3 (METTL3) and methyltransferase-like protein 14 (METTL14), as well as demethylases, such as fat mass and obesity–associated protein (FTO) and AlkB homolog 5 (Yang et al., 2018). m6A modification also participates in physiological processes, such as synaptic plasticity, circadian rhythm, and stress response, in the nervous system (Chokkalla et al., 2020). The maladjustment of m6A modification is connected to a variety of neurological diseases, such as stroke, peripheral nerve injury, and Parkinson’s disease (Han et al., 2020; Weng et al., 2018; Yu et al., 2020).

## 3 Epigenetic modification plays a role in NP information processing

Neuropathic pain resulting from nerve injury is strongly associated with alterations in epigenetic modifications. These modifications dynamically regulate the expression of pain-related genes, such as those encoding ion channels, neurotransmitter receptors, and inflammatory mediators, leading to the sensitization of the peripheral and central nervous systems. This process contributes to the establishment and maintenance of a persistent chronic pain state. Such epigenetic mechanisms offer a molecular basis for how environmental insults can be converted into long-lasting pain “memory.” Notably, these epigenetic mechanisms do not function independently. Instead, they collectively form a highly integrated regulatory network that acts synergistically to modulate gene expression. For example, m6A modification can indirectly regulate DNA methylation patterns through its influence on the enzymes involved in DNA methylation (Pilala et al., 2025). Moreover, accumulating evidence demonstrates that non-coding RNAs function as molecular scaffolds to facilitate the direct recruitment of histone-modifying enzymes to defined genomic loci, whereas miRNAs exert their regulatory effects by posttranscriptionally suppressing the mRNA of these enzymes, thereby indirectly modulating histone modification patterns (Hemphill et al., 2023; Mortazavi et al., 2022). Furthermore, DNA methylation, particularly within gene promoter regions, frequently collaborates with repressive histone modifications to establish a condensed chromatin architecture that effectively silences specific genes, including those involved in analgesia. By contrast, active histone modifications are commonly associated with hypomethylated genomic regions and facilitate gene expression (Yakhnitsa et al., 2024; Yuan et al., 2022). Although this review primarily centers on the relationship between a specific epigenetic modification and NP, the aforementioned evidence underscores the necessity of developing a comprehensive understanding of how multiple epigenetic mechanisms interact and collectively influence NP pathogenesis.

### 3.1 DNA methylation and NP

A remarkable characteristic of NP is the chronic overactivity of damaged dorsal root ganglion (DRG) sensory neurons depending on constant changes in the expression levels of numerous genes (Guo et al., 2021). DNA methylation can directly affect pain sensibility and occurs at cytosine in CpG dinucleotides (Zhao et al., 2020). Through simplified methylation sequencing, Garriga et al. (2018) conducted the whole-genome detection of damaged DRG tissue in peripheral nerves injured for 3 weeks. They found that 1083 (6.5%) CpG sites appeared to be in a hypomethylated state, whereas 227 (1.4%) were in a hypermethylated state. Combined mRNA transcriptome sequencing analysis revealed consistent changes in the levels of the expression (fold change > 2, *p* < 0.05) and DNA methylation (>2%, *p* < 0.05) of 664 genes. The increase in variable gene expression was related to different methylation regions after peripheral nerve injury; the variable methylation of CpG near the start of transcription was associated with the reduction in gene expression. Moreover, the DNA methylation of DRG after peripheral nerve injury was closely associated with changes in gene expression. DNA methylation usually restrains gene expression, and demethylation re-expresses genes (Greenberg and Bourc’his, 2019). Chao et al. (2016) found that the considerable demethylation in CpG islands in the promoter region of the brain-derived neurotrophic factor (BDNF) gene in DRG can improve BDNF expression and participate in NP. DNA methylation is catalyzed by DNA methyltransferase (DNMT). Injury to the peripheral neuron system increases the expression of DNMT3 in injured DRG neurons. However, terminating the increased expression of DNMT3a can prevent the methylation of the voltage-dependent potassium channel subunit (Kcna2) promoter region caused by nerve injury and restore the expression of Kcna2 in damaged DRG, thereby alleviating NP (Zhao et al., 2020). DNA methylation can directly regulate the expression of specific genes in DRG. Moreover, the expression of DNMT3b decreased in the spinal neurons of mice after spinal nerve ligation (SNL). This effect led to the demethylation of the chemokine receptor 3 (CXCR3) promoter and promoted the combination of CCAAT/C/EBPα with the CXCR3 promoter. It further improved the expression of CXCR3 in spinal neurons. The upregulation of CXCR3 may promote NP through central sensitization (Jiang et al., 2017). Additional relevant studies further corroborate the aforementioned perspective. Specifically, in neonatal rats, repeated neonatal surgical pain has been linked to elevated DNA methylation levels in the μ-opioid receptor (MOR) promoter within the spinal cord. Moreover, the hypermethylation of the membrane-bound catechol-*O*-methyltransferase promoter coupled with reduced IFN-γ expression represents a distinctive molecular signature in patients diagnosed with chronic fatigue syndrome and fibromyalgia (Baudat et al., 2025; Polli et al., 2022).

Changes in DNA methylation in blood can be a feature of specific changes in the brain or other tissues. The analysis of the function of genes in different methylation regions showed that nociceptive pain was associated with the function of the opioid analgesic system, and NP was related to the function of the GABAergic rewards system (Stenz et al., 2022). Through genome-wide DNA sequencing, research distinguished nociceptive pain from NP to identify different methylation features in the blood of patients with nociceptive pain and NP.

### 3.2 Non-coding RNAs and NP

microRNAs can regulate mRNA expression at the posttranscriptional level and participate in the regulation of NP pathways. Chen M. et al. (2020) found that in mice with nerve injury, the expression of miR-154-5P increased in the spinal cord and that of the CXCL13 protein was inhibited. In addition, markedly increasing lncRNA SNHG5 expression in the spinal cord of mice and inhibiting SNHG5 could increase the expression of miR-154-5p. Finally, in mice, knocking out SNHG5 revealed that the lack of the lncRNA SNHG5 could inhibit the activation of astrocytes and microglia through the miR-154-5p/CXCL13 axis and then alleviated NP symptoms. Zhang et al. (2021) found that the absence of circ0005075 can ameliorate NP by inducing the inactivation of miR-151a-3p and NOTCH2 signaling, and NOTCH2 could induce various intracellular reactions. These lines of evidence indicate that non-coding RNAs participate in the pathogenesis of NP and can be the precision regulator of NP-specific gene expression. Therefore, non-coding RNAs have considerable potential as peripheral biomarkers for the clinical diagnosis and monitoring of the treatment efficacy of NP. Research on the biomarkers of NP has entered a new stage with the development of high-throughput sequencing technology and improvement in big data computing power. In clinical research, Ye et al. (2021) compared patients with NP caused by spinal cord injury with healthy people and collected their sera for whole-genome miRNA expression profile screening. They found that differential miRNA expression verification results and serum has-miR-19a-3p and has-miR-19b-3p could easily distinguish patients with pain from healthy individuals. Tramullas et al. (2018) reported that in NP mice, sciatic nerve injury contributes to the upregulated expression of miR-30c-5p in the DRG, spinal cord, cerebrospinal fluid, and plasma, with the expression of miR-30c-5p being positively correlated with the severity of hypersensitivity to pain. Researchers found that miR-30c-5p inhibitors in the cisterna could stop the development of NP and completely reverse hypersensitivity to pain. In a clinical trial, researchers chose 25 patients with NP due to chronic peripheral ischemia and compared them with those without pain. They discovered that the expression of miR-30c-5p significantly increased in the plasma and cerebrospinal fluid of patients with NP. Logistic regression analysis showed that the increased expression of miR-30c-5p in plasma and cerebrospinal fluid may help predict the occurrence of NP in patients with chronic peripheral ischemia. Yu et al. (2017) recruited 154 patients with NP induced by type 2 diabetes to explore the role of the lncRNA NON-RATT021972 in NP. The concentration of the lncRNA NON-RATT021972 in the blood of patients with type 2 diabetes had significantly increased compared with that in the control group and was positively correlated with pain score. However, the researchers did not observe any associations between neuropathy and the lncRNA NON-RATT021972 in patients who had no diabetes but had neuropathy. Cao et al. (2019) discovered that the miRNA and circRNA expression profiles in the skin of patients with postherpetic neuralgia were markedly altered relative to those in unaffected mirror skin. They found 317 differentially expressed miRNAs and 31 circRNAs. These findings have important guiding value for the early diagnosis and effective treatment of NP.

### 3.3 Histone modification and NP

A large body of evidence shows that changes in histone acetylation are the reasons for the induction and maintenance of NP. Key enzymes in histone acetylation include histone deacetylases (HDACs) and histone acetyltransferases (HATs). Research suggests that nerve injury upregulates HDACs, leading to increased histone deacetylation, inhibited related gene expression, and eventually to NP. Li et al. (2019) explored which HDAC subunit regulates Kv1.2 expression and participates in the development of NP. Their immunofluorescence results illustrated that Kv1.2 colocated with HDAC2 in DRG macroneurons but not with HDAC1. In mice with chronic sciatic nerve compression injury (CCI), the intrathecal injection of HDAC inhibitors alleviated mechanical and thermal anaphylaxis and reversed the decline in Kv1.2 expression. In *in vitro* experiments, PC12 cells were transfected with HDAC2 and HDAC1 siRNA, and only HDAC2 siRNA was found to regulate Kv1.2 expression. In conclusion, HDAC2 regulates the expression of Kv1.2 and then participates in the development of NP. Ouyang et al. (2019) found that HDAC2 mRNA and protein levels in the spinal cord of CCI rats were markedly elevated compared with those in rats that underwent sham surgery. Their research confirmed that HDAC2 mediated mechanical and thermal pain caused by peripheral nerve injury. The sirtuin family is a group of nicotinamide adenine dinucleotide– dependent deacetylases that modulate various physiological and pathological processes by deacetylating histones and other specific substrates. In CCI mice, decreased SIRT1 and SIRT2 deacetylase activities may be a factor (Shao et al., 2014; Zhang and Chi, 2018) in promoting the development of NP. A few works have reported that HAT expression increased after nerve injury; this phenomenon promoted histone acetylation, enhanced the transcription of related genes, and finally induced NP. Kiguchi et al. (2012) found that in some mouse models of sciatic nerve ligation, histone H3K9 in the MIP-2 and CXCR2 promoter region was upregulated in damaged sciatic nerves. HAT inhibitors inhibit the upregulation of MIP-2 and CXCR2 in damaged sciatic nerves, thereby preventing partial sciatic nerve injection–induced NP. p300 and its homolog CBP are two functionally related proteins in the HAT family that participate in numerous biological processes, such as neurodevelopment and cognition. Zhu et al. (2014) found that the manifestation of NP induced by CCI was associated with the increased expression of P300/CBP in the dorsal horn of the spinal cord in mice, and curcumin alleviated NP by downregulating the expression of the BDNF and Cox-2 genes. This downregulation was mediated byp300/CBP HAT activity. Current animal experiments suggest that histone modifications have the theoretical ability to alter peripheral and central pain pathways.

### 3.4 mRNA modification and NP

In recent years, evidence has shown that m6A modification is associated with the occurrence and maintenance of NP. In Li Y. et al. (2020) demonstrated the role of m6A modification in NP and detected the expression of m6A methyltransferase, demethylase, and m6 binding protein in the L5 DRG on the injured side of SNL mice. The mRNA and protein expression levels of the demethylase FTO in L5 DRG on the injured side had significantly increased compared with those on the healthy side. The differences in the expression levels of the m6A methyltransferases METTL3 and METTL14 and binding proteins WTAP and YTHDF2 were not statistically significant. The researchers also compared different NP models and found that although the results of the CCI model were similar to those of the SNL model, the NP model induced by the subplantar injection of complete Freund’s adjuvant (CFA) did not exhibit significant changes in FTO protein levels. FTO is a member of the N6-methyladenosine demethylase group and mainly functions in clearing m6A from RNA. By using m6A-enhanced purple immunoprecipitation combined with high-throughput sequencing technology, the researchers found that approximately 56% of RNAs in DRG neurons after peripheral nerve injury had m6A site loss, with Ehmt2 mRNA showing the most significant m6A site loss. Ehmt2 mRNA can encode the G9a protein. Further research confirmed that after peripheral nerve injury, the high expression of FTO in DRG neurons can upregulate G9a and downregulate MOR. Moreover, the inhibition of FTO can reverse the loss of m6A sites from Ehmt2 mRNA, stop the upregulation of G9a, and reverse the downregulation of MOR. These results illustrated that FTO inhibitors can not only reduce NP but also delay the development of morphine tolerance in NP. Zhang et al. (2022) detected DNMT METTL3 m6A methylation in the spinal cord of SNL mice. METTL3 and m6A methylation expression in the spinal cords of NP mice were significantly downregulated relative to those in sham operation mice. Meanwhile, METTL3 upregulation could promote m6A methylation in total RNA and inhibit NP progression. However, METTL3 silencing inhibited m6A methylation and aggravated NP. Further research has shown that METTL3/YthDF2-mediated m6A modification regulates NP development by regulating miR-150 and inhibiting BDNF expression. In addition, in clinical trials, METTL3 mRNA was downregulated in the serum of patients with herpes zoster and NP, showing the potential of METTL3 as a diagnostic biomarker. In addition, FTO participates in NP-induced anxiety and depression. The expression of FTO in the anterior cingulate cortex of mice is downregulated after peripheral nerve injury, thereby inhibiting the expression of MMP-9, reducing the level of mBDNF, and inducing anxiety and depression-like behavior (Wang et al., 2022). FTO may be an endogenous trigger of NP-induced anxiety and depression-like behavior. The above finding provides potential targets for the development of new treatments.

## 4 Epigenetic mechanisms of exercise in NP

**FIGURE 1:**
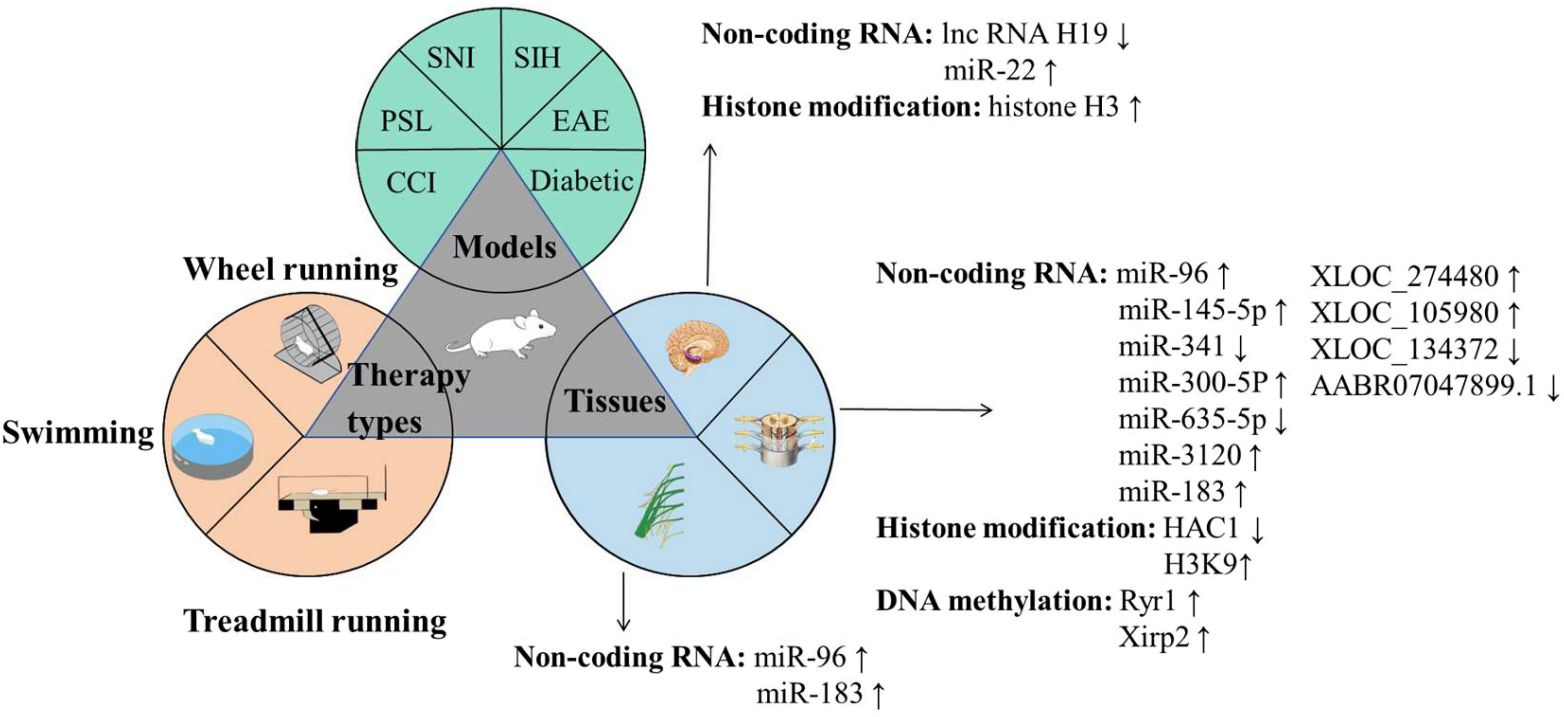
Key elements underlying epigenetic participating in exercise-relieving NP.

Exercise is closely associated with the improvement in NP and can induce beneficial physiological and biochemical changes in patients with NP (Zheng et al., 2024). Regular exercise can influence the epigenetic modifications of the body through various mechanisms, including methylation and histone modification (Bittel and Chen, 2024; Massart et al., 2021; Shimizu and Kawano, 2022). We conducted a systematic review focusing on animal studies to identify the key epigenetic mechanisms underlying the exercise-induced improvement in NP. The following inclusion criteria were defined: (1) studies using established NP models (e.g., CCI, diabetic neuropathy, and SNL); (2) clearly defined exercise interventions (e.g., swimming and treadmill running) with specified parameters; and (3) epigenetic analyses performed on pain-related tissues (e.g., DRG, spinal cord, and sciatic nerve). We excluded reviews, conference abstracts, methodological papers, and publications in languages other than English or Chinese. Our search strategy employed a combination of MeSH terms and keywords related to neuropathic pain, epigenetics, and exercise across PubMed, Web of Science, and CNKI from inception to August 2025 (Supplementary File 1). After we performed deduplication in EndNote X9, two trained researchers (YX and HS) independently screened records by title and abstract. Full-text assessment was then conducted. Discrepancies were resolved through discussion or third-party arbitration (JG). Finally, we performed the backward citation tracking of the included studies and relevant reviews to ensure comprehensive coverage. Finally, eight articles and four theses examining epigenetic regulatory mechanisms in exercise-mediated NP improvement were included (Aghdam et al., 2018; Chen et al., 2025; Guo et al., 2021; Imbe and Kimura, 2015, 2016; Kakavandi et al., 2025; Kami et al., 2016; Song et al., 2022; Su, 2022; Xu et al., 2024; Yang, 2021; Zheng, 2022). Study selection is illustrated in Supplementary File 2. Figure 1 and Table 1 show the results of the NP models, exercise modalities, sampling tissues, and epigenetic modifications in the included research.

**TABLE 1:** Characterization of the included literatures.

Trial	Country	Tissues	NP model	Sample size	Therapy type	Exercise duration	Exercise frequency	Types of epigenetic modifications
Kami et al., 2016	Japan	Spinal cord	PSL	Naive (*n* = 4), Sham-sedentary (*n* = 4), Sham-runner (*n* = 4), PSL-sedentary (*n* = 6), PSL-runner (*n* = 6)	Treadmill	3 weeks	5 days/week	Histone modification: HDAC1 and H3K9
Su, 2022	China	Hippocampus	SNI	Naive (*n* = 12), Sham (*n* = 12), Sham + swim (*n* = 12), SNI (*n* = 12), SNI + swim (*n* = 12)	Swimming	6 weeks	6 days/week	Non-coding RNA: lnc RNA H19 and miR-22
Yang, 2021	China	DRG	CCI	Sham (*n* = 12), CCI (*n* = 12), CCI + swim (*n* = 12)	Swimming	4 weeks	5 days/week	Non-coding RNA: miR-96
Guo et al., 2021	China	DRG	CCI	CCI (*n* = 6), Sham (*n* = 6), CCI + swim (*n* = 6)	Swimming	4 weeks	6 days/week	Non-coding RNA: miR-145-5p, miR-341, miR-300-5p and miR-653-5p
Song et al., 2022	China	Spinal cord	CCI	Sham (*n* = 6), CCI (*n* = 6), CCI + swim (*n* = 6)	Swimming	4 weeks	6 days/week	Non-coding RNA: XLOC_274480, XLOC_105980, XLOC_134372 and AABR07047899.1
Aghdam et al., 2018	Iran	Sciatic nerve	Diabetic	Control group (*n* = 12), Swimming (*n* = 12), Diabetic group (*n* = 12), Diabetic + swim (*n* = 12)	Swimming	10 weeks	5 days/week	Non-coding RNA: miR-96
Imbe and Kimura, 2016	Japan	RVM and LC	SIH	Naive (*n* = 8), CFA (*n* = 8), FS (*n* = 7), CFA + FS (*n* = 8)	Swimming	7–8 days	For 10 min on day 1, for 20 min on days 2–3	Histone modification: histone H3
Imbe and Kimura, 2015	Japan	IC and ACC	SIH	Naive (*n* = 8), CFA (*n* = 20), FS (*n* = 19), CFA + FS (*n* = 20)	Swimming	12 days	For 10 min on day 1, for 20 min on days 2–3	Histone modification: histone H3
Xu et al., 2024	China	DRG	SIH	Naive (*n* = 6), FS (*n* = 6), CFA (*n* = 6), CFA + FS (*n* = 6)	Swimming	3 days	For 10 min on day 1, for 20 min on days 2–3	Non-coding RNA: miR-3120
Zheng, 2022	China	Sciatic nerve and DRG	SNI	Naive (*n* = 15), Shame (*n* = 15), Shame + swim (*n* = 15), SNI (*n* = 15), SNI + swim (*n* = 15)	Swimming	6 weeks	Exercise for 3 days and rest 1 day	Non-coding RNA: miR-183
Chen et al., 2025	China	DRG	CCI	CCI (*n* = 6), Sham (*n* = 6), CCI + swim (*n* = 6)	Swimming	6 weeks	5 days/week	DNA methylation: Ryr1, Xirp2
Kakavandi et al., 2025	Iran	DRG	EAE	EAE (*n* = 6) Health (*n* = 6) Resistance exercise + EAE (*n* = 6) Aerobic exercise + EAE (*n* = 6)	Exercise	4 weeks	5 days/week	Non-coding RNA: miR-155

ACC, anterior cingulate cortex; CCI, chronic constriction injury; CFA, complete Freund’s adjuvant; DRG, dorsal root ganglion; EAE, experimental autoimmune encephalomyelitis; FS, swimming exercise; HDAC1, histone deacetylase 1; H3K9, histone H3 lysine 9; IC, insular cortex; lnc RNA H19, long non-coding RNA H19; LC, locus coeruleus; miR, microRNA; PSL, partial sciatic nerve ligation; RVM, rostral ventromedial medulla; Ryr1, ryanodine receptor 1; SIH, sural and inferior plantar nerve injury; SNI, spared nerve injury; Xirp2, Xin actin-binding repeat containing protein 2.

### 4.1 Exercise alters nP-related non-coding RNAs

We retrieved seven studies on the mechanism of non-coding RNAs in exercise treatment to improve NP (Aghdam et al., 2018; Guo et al., 2021; Song et al., 2022; Su, 2022; Xu et al., 2024; Yang, 2021; Zheng, 2022). Our previous research found that in CCI mice, the adaptation mechanism of DRG tissue to exercise may be associated with the expression of different miRNAs. Transcriptome sequencing and bioinformatics revealed that miR-145-5p, miR-341, miR-300-5p, and miR-653-5p may be new targets in the therapeutic mechanism of exercise as an intervention for NP. Cell and animal experiments demonstrated that in CCI mice, miR-145-5p may participate in the therapeutic mechanism (Guo et al., 2021) of exercise to improve pain hypersensitivity by targeting the inhibition of Cacna2d1 expression. Cacna2d1 is a voltage-gated calcium channel subunit and is an important mechanism in the induction and maintenance of NP. Exercise can alter miRNA expression levels, and miRNA may alleviate NP symptoms by regulating pain-related target genes. Similar experiments were also conducted by Aghdam et al. (2018) and Yang (2021), who explored the influence of swimming on the expression of miR-96 in NP mice. Multiple studies suggest that miR-96 negatively regulates the expression of Nav1.3 (encoded by the SCN3A gene), and this regulatory relationship is implicated in NP. Aghdam et al. (2018) found that compared with those in the control group, the expression of miR-96 in the sciatic nerve of mice with diabetic peripheral neuropathy significantly decreased, whereas the expression of NaV 1.3 was significantly upregulated. After 10 weeks of swimming, the expression of miR-96 recovered and that of NaV 1.3 significantly decreased. Yang (2021) used DRG and found that the expression of miR-96 in CCI mice decreased significantly, whereas the expression levels of NaV1.3, Cacna2d1, Cacna2d2, TNF-α, and IL-6 increased significantly. Four-weeks swimming training reversed the expression of miR-96. The expression levels of NaV 1.3, Cacna2d1, Cacna2d2, TNF-α, and IL-6 decreased significantly. In addition, Xu et al. (2024) examined the role of the heat-shock cognate 71 kDa protein (Hsc70) and its regulatory factor miR-3120 in forced swimming stress (FS)–induced mechanical hyperalgesia in rats with inflammatory conditions. FS significantly exacerbated the mechanical pain induced by CFA. The expression of miR-3120 significantly increased on the third day post-FS, and miR-310 colocalized with Hsc70, showing expression in DRG neurons. The injection of miR-3120 agomir into DRG led to TRPV1 expression and behavioral changes akin to those induced by FS stress. These findings suggest that miR 3120/Hsc70 may play a role in FS stress–induced mechanical hyperalgesia in rats in an inflammatory state.

Song et al. (2022) explored the effect of swimming on lncRNAs in the L4–L6 spinal dorsal horn on the injured side of CCI mice. Behavioral test results showed that compared with those in the no-exercise group, CCI mice in the exercise group showed significant improvements in mechanical and thermal hyperalgesia symptoms. High-throughput sequencing combined with bioinformatics analysis demonstrated that 385 lncRNAs were upregulated and 349 lncRNAs were downregulated in the CCI group relative to those in the sham operation group. Comparison between the exercise and CCI groups revealed that 339 lncRNAs were upregulated and 419 lncRNAs were downregulated. The results showed 306 overlapping lncRNAs in the two combinations. The researchers verified four lncRNAs by RT-PCR. These lncRNAs were XLOC_274480 and XLOC_105980 in the comparison between the CCI and sham operation groups and XLOC_137372 and AABR07047899.1 in the comparison between the exercise and CCI groups.

### 4.2 Exercise alters nP-related histone modification

Three studies investigated the mechanism of histone modification during exercise (Imbe and Kimura, 2015, 2016; Kami et al., 2016) to improve the efficacy of its mechanism in NP treatment. Kami et al. (2016) established a partial sciatic nerve ligation mouse model and subjected the model to 60% maximal oxygen consumption at 2 days after surgery for 60 min a day for 5 days a week. After 3 weeks of exercise, the symptoms of mechanical stimulation and thermal hyperalgesia in the mice in the exercise group had significantly reduced relative to those in the mice in the non-exercise group. The immunofluorescence experiment proved that HDAC1 immune response occurred in the superficial dorsal horn of the spinal cord on the injured side of the model mice but weakened after exercise. HDAC1 mainly existed in microglia. Histone acetylation may also be affected, and HDAC1 is an important enzyme that regulates H3K9 acetylation. The results also confirmed that the expression of histone H3K9 decreased after injury and significantly increased after running. In summary, the acetylation of H3K9 in activated microglia may play a key role in the reduction in NP by exercise.

Imbe and Kimura (2015, 2016) assessed phospho-cAMP response element-binding protein (pCREB), ΔFosB, and histone H3 acetylation in the rostral ventromedial medulla (RVM), locus coeruleus (LC), insula (IC), and anterior cingulate cortex (ACC) following FS exposure and CFA administration. FS significantly elevated H3 acetylation in the RVM and LC. CFA administration following FS exacerbated mechanical hyperalgesia in rats and negated heightened histone H3 acetylation and pCREB and ΔFosB expression levels in LC and IC but did not influence FS-induced acetylation in the RVM. This study further confirmed that an increase in histone acetylation is associated with the activation of gene transcription and that histone acetylation plays a crucial role in pain perception and behavioral adaptation during chronic stress (Khangura et al., 2017).

### 4.3 Exercise alters nP-related DNA methylation and mRNA

Recently, a groundbreaking study combining transcriptomic and whole-genome bisulfite sequencing analyses established the first causal connection between exercise-induced DNA methylation remodeling in the DRG and the alleviation of NP. Chen et al. (2025) showed that 6 weeks of swimming-based prehabilitation prior to sciatic chronic constriction injury significantly reduced postoperative mechanical and thermal hyperalgesia in rats. This protective effect was correlated with 396 differentially methylated regions in the L4-L6 DRGs of injured rats. Integrated methylomic and transcriptomic profiling revealed the hypermethylation of promoter regions in the calcium-release channel gene Ryr1 and actin-binding gene Xirp2. This effect coincided with the increased mRNA expression levels of these genes in CCI rats. Notably, exercise prehabilitation specifically reversed the hypermethylation of Ryr1, thereby restoring its abnormal expression to baseline levels. However, no additional relevant studies that specifically aim to clarify the mechanistic roles of DNA methylation and mRNA modifications in the context of exercise intervention for NP have been identified to date. Therefore, the potential biological mechanisms underlying exercise-induced DNA methylation and mRNA modifications in NP require further investigation and comprehensive analysis within an expanded systems biology framework.

#### 4.3.1 Potential mechanisms of exercise altering DNA methylation

We reviewed interventional research on the modification of DNA methylation by exercise and found that exercise can induce changes in DNA methylation status in the nervous system. Etayo-Urtasun et al. (2024) conducted a systematic review following PRISMA 2020 guidelines to explore the effects of exercise on DNA methylation patterns. They retrieved 12 randomized controlled trials involving 827 previously inactive adults from PubMed, Scopus, and Web of Science, with intervention durations ranging from 6 weeks to 12 months, and evaluated methodological quality by using the PEDro scale. Their results showed that most trials confirmed that exercise interventions could significantly alter the DNA methylation of specific genes (e.g., RANKL, FKBP5, and AURKA) and global DNA methylation patterns, with the increased methylation of key genes and decreased global methylation being observed. The heterogeneity of findings was attributed to differences in participant demographics, intervention factors, measurement techniques, and genomic contexts. The authors concluded that future research should focus on analyzing the influences of exercise type, intensity, and duration, clarifying dose– response relationships and identifying exercise-responsive genes to deepen the understanding of the molecular mechanisms underlying the role of exercise in disease prevention and treatment. Although human studies, as summarized in the meta-analysis, have focused on skeletal muscle and highlighted exercise-induced DNA methylation changes in genomic localization, transcriptional associations, and influencing factors, basic animal research has further demonstrated that exercise can alter DNA methylation in specific neural tissues with implications for neural function and pain modulation.

The evidence, encompassing human and animal studies, published over the past decade has predominantly focused on skeletal muscle and has highlighted alterations in the genomic localization, transcriptional associations, and modulating factors of exercise-induced DNA methylation (Bittel and Chen, 2024). However, animal research further indicates that exercise can modify DNA methylation patterns in specific neural tissues, including the hippocampus, cortex, motor cortex, and DRG, potentially influencing neural function and pain regulatory mechanisms. Rodrigues et al. (2015) found that 4 weeks of exercise can alter the whole DNA methylation status of the hippocampus, cortex, and periaqueductal gray matter. Ferrari et al. (2019) discovered that exercise has a significant effect on DNA methylation levels in some cells. Davaa et al. (2021) reported that in mice with spinal cord injury, 12 weeks of exercise can enhance DNA methylation in the motor cortex, then promoted the recovery of motor function. In addition, Barrès et al. (2012) biopsied the lateral femoris muscle after exercise and found a reduction in whole DNA methylation levels. Kawarai et al. (2021) used voluntary exercise to intervene in model mice with back pain induced by disk degeneration and measured the change in overall DNA methylation level in the disks of the mice after 6 months of exercise. Their results are consistent with those of Barrès et al. (2012). Back pain was significantly relieved and DNA methylation levels in the intervertebral disks of the mice in the exercise group were significantly reduced compared with those in the inactive group. Although basic research supports that exercise can change the level of DNA methylation in the nervous system and DNA methylation participates in the occurrence and development of NP, direct evidence is limited with regard to the involvement of DNA methylation in the mechanism of exercise to improve NP.

#### 4.3.2 Potential mRNA-altering mechanisms of exercise

Furthermore, limited research has been conducted on the effect of exercise on mRNA modification. Nevertheless, exercise, as a physiological stimulus, may modulate mRNA modification processes through alterations in intracellular signaling pathways and metabolic conditions. Accumulating evidence indicates that exercise can influence the mRNA expression levels of genes involved in energy metabolism, skeletal muscle adaptation, and neural function. A study (Danaher et al., 2020) examined the influence of high-intensity versus low-intensity exercise on FTO mRNA and protein expression levels in individuals with a healthy body weight. Its results demonstrated that high-intensity exercise markedly reduced FTO mRNA expression. No considerable differences in FTO protein levels were observed at baseline or following exercise across different FTO genotypes. These findings suggest that beyond its regulation by nutritional factors, the FTO gene may be influenced by physical exercise. Through RNA sequencing and qRT-PCR verification, Liu et al. (2022) found that FTO expression in the hippocampus and hypothalamus of significantly decreased but m6A expression significantly increased in the mice in the 12-weeks treadmill exercise group relative to in the non-exercise group. They hypothesized that long-term exercise might increase the level of m6A-labeled transcripts in the hippocampus and hypothalamus by downregulating FTO. Yan et al. (2022) found that exercise can promote the recovery of m6A in the medial prefrontal cortex of mice, and increasing its activity can play an antianxiety role. The aforementioned studies confirmed that physical exercise can influence mRNA modification processes across multiple physiological systems, including the nervous system. However, to date, no research has directly elucidated the specific mechanism through which exercise alleviates NP via the regulation of mRNA modification.

The three circles represent the three major categories of research content in this field: The green circle illustrates the commonly used animal models, including SNI, SIH, CCI, PSL, and Diabetic models; The yellow circle depicts the frequently employed exercise intervention protocols, such as voluntary wheel running, swimming training, and treadmill training; The blue circle highlights the key tissue targets of investigation, encompassing the peripheral sciatic nerve, dorsal root ganglion (DRG), spinal dorsal horn (SDH), and specific brain regions (RVM, LC, IC, ACC, and hippocampus). With regard to molecular mechanisms, brain tissue studies primarily focus on IncRNA H19, miR-22, and histone H3; investigations involving the spinal dorsal horn and DRG center on miR-96, miR-145-5p, miR-341, miR-300-5p, miR-653-5p, miR-3120, miR-183, XLOC_274480, XLOC_105980, XLOC_1134372, AABR07047899.1, as well as HDAC1 and H3K9 modifications; while miR-96 and miR-183 are predominantly studied in relation to the sciatic nerve. Abbreviations: ACC, Anterior cingulate cortex; CCI, Chronic constriction injury; DRG, Dorsal root ganglion; EAE, Experimental autoimmune encephalomyelitis; HDAC1, Histone deacetylase 1; H3K9, Histone H3 lysine 9; IC, Insula; LC, Locus coeruleus; lncRNA, Long non-coding RNA; miRNA, microRNA; NP, Neuropathic pain; PSL, Partial sciatic nerve ligation; RVM, Rostral ventromedial medulla; SNI, Spared nerve injury; SIH, Stress-induced hyperalgesia.

## 5 Conclusion

Few studies have been conducted on the direct association among exercise, NP, and epigenetic modification. Exercise can indeed improve NP by altering non-coding RNAs and histone modification, providing a new concept for endogenous regulatory mechanisms. Epigenetics responds to exercise-mediated changes in gene expression and provides NP biomarkers. It not only helps expound the potential mechanism of exercise therapy for NP but also provides quantifiable biological indicators for the clinical evaluation of NP treatment efficacy. Although exercise-based mechanisms can improve NP, the specific pathways through which exercise can regulate epigenetic mechanisms to ameliorate NP remain to be studied. In addition, the evidence accumulated in basic experiments should be transformed into clinical studies in the future. The understanding of the epigenetic mechanisms of exercise analgesia should then be continuously improved. Moreover, a theoretical foundation for the further comprehension of the mechanisms of exercise analgesia should be provided.
