# Supplementary material for: Epigenetic remodeling: unveiling the potential endogenous mechanisms of exercise in alleviating neuropathic pain
Source: Front Neurosci. 2025 Sep 5;19:1622894. doi: 10.3389/fnins.2025.1622894 (PMC12446320; doi:10.3389/fnins.2025.1622894)
Supplement: Supplementary file 1 [file Table_1.docx]

**Search strategy**

| **1. CNKI** |  |
| --- | --- |
| #1 (TKA='神经病理性疼痛' OR TKA='神经病理性痛' OR TKA='神经痛' OR TKA='糖尿病神经病变')  #2 (TKA='神经损伤' OR TKA='神经结扎' OR TKA='神经炎症') AND (TKA=疼痛)  #3 #1 OR #2  #4 (TKA='表观遗传' OR TKA='DNA甲基化' OR TKA='非编码RNA' OR TKA='MicroRNA' OR TKA='miRNA' OR TKA='微小RNA' OR TKA='长链非编码RNA' OR TKA=' lncRNA' OR TKA='环状RNA' OR TKA='circRNA')  #5 (TKA='运动' OR TKA='跑步' OR TKA='跑台' OR TKA='游泳' OR TKA='丰富环境' OR TKA='转轮'')  #6 #3 AND #4 AND #5 |  |
| **2. PubMed** | |
| #1. Neuralgia[Mesh] OR Neuralgia*[Title/Abstract] OR Neurodynia*[Title/Abstract] OR "Neuropathic pain*"[Title/Abstract] OR “Nerve pain*”[Title/Abstract] OR sciatica[Title/Abstract] | |
| #2. "nerve crush"[Title/Abstract] OR "nerve cut"[Title/Abstract] OR "nerve constriction"[Title/Abstract] OR "nerve inflammation"[Title/Abstract] OR "nerve injury"[Title/Abstract] OR "nerve ligation"[Title/Abstract] OR "peripheral neuropathy"[Title/Abstract] OR "chronic constriction injury"[Title/Abstract] OR "diabetic neuropathy"[Title/Abstract] OR pre-diabetes[Title/Abstract] OR "metabolic syndrome"[Title/Abstract] OR "high-fat diet"[Title/Abstract] OR diet-induced[Title/Abstract] OR [chemotherapy-induced[Title/Abstract]](https://www-ncbi-nlm-nih-gov-nus.vtrus.net/pubmed/30470691) OR "paclitaxel-induced"[Title/Abstract] OR streptozotocin-induced[Title/Abstract] | |
| #3. pain*[Title/Abstract] | |
| #4. #2 AND #3 | |
| #5. #1 OR #4 | |
| #6. Exercise[Mesh] OR "Exercise therapy"[Mesh] OR Locomotion[Mesh] OR exercise*[Title/Abstract] OR locomotion[Title/Abstract] OR running[Title/Abstract] OR swim*[Title/Abstract] OR "environmental enrichment"[Title/Abstract] OR treadmill[Title/Abstract] OR vibration[Title/Abstract] OR aerobic*[Title/Abstract] OR strength*[Title/Abstract] OR isometric*[Title/Abstract] OR isotonic*[Title/Abstract] OR isokinetic*[Title/Abstract] OR endurance[Title/Abstract] OR weight*[Title/Abstract] OR physiotherapy[Title/Abstract] OR resistance[Title/Abstract] OR train*[Title/Abstract] | |
| #7 epigenetic*[Title/Abstract] OR methylation[Title/Abstract] OR histone OR acetylation[Title/Abstract] OR acetyltransferase[Title/Abstract] OR methyltransferase[Title/Abstract] OR deacetylases[Title/Abstract] OR HDAC*[Title/Abstract] OR HAT*[Title/Abstract] OR MicroRNA*[Title/Abstract] OR mir*[Title/Abstract] OR "micro RNAs"[Title/Abstract] OR "micro RNA"[Title/Abstract] OR micro-RNAs[Title/Abstract] OR micro-RNA[Title/Abstract] OR lncRNA*[Title/Abstract] OR "long ncRNA*"[Title/Abstract] OR "long noncoding RNA*"[Title/Abstract] OR circRNA*[Title/Abstract] OR "Circular RNA*"[Title/Abstract] | |
| #8. #5 AND #6 AND #7 | |
| **3. Web of science** | |
| #1 TS=(Neuralgia* OR Neurodynia* OR "Neuropathic pain*" OR sciatica) | |
| #2 TS=("nerve crush" OR "nerve cut" OR "nerve constriction" OR "nerve inflammation" OR "nerve injury" OR "nerve ligation" OR "peripheral neuropathy" OR "chronic constriction injury" OR "diabetic neuropathy" OR pre-diabetes OR "metabolic syndrome" OR "high-fat diet" OR diet-induced OR chemotherapy-induced OR "paclitaxel-induced" OR streptozotocin-induced) | |
| #3. TS=(pain*) | |
| #4. #2 AND #3 | |
| #5. #1 OR #4 | |
| #6. TS=(exercise* OR locomotion OR run* OR swim* OR "environmental enrichment" OR treadmill OR vibration OR aerobic* OR strength* OR isometric* OR isotonic* OR isokinetic* OR endurance OR weight* OR physiotherapy OR resistance OR train*) | |
| #7. TS=(epigenetic* OR methylation OR histone OR acetylation OR acetyltransferase OR methyltransferase OR deacetylases OR HDAC* OR HAT* OR MicroRNA* OR mir* OR "micro RNAs" OR "micro RNA" OR micro-RNAs OR micro-RNA OR lncRNA* OR "long ncRNA*" OR "long noncoding RNA*" OR circRNA* OR "Circular RNA*") | |
| #8. #5 AND #6 AND #7 | |
| Timespan=All years. Databases=SCI-EXPANDED. Article Type= Article | |
