# Supplementary figures and images for: Epigenetic remodeling: unveiling the potential endogenous mechanisms of exercise in alleviating neuropathic pain
Source: Front Neurosci. 2025 Sep 5;19:1622894. doi: 10.3389/fnins.2025.1622894 (PMC12446320; doi:10.3389/fnins.2025.1622894)

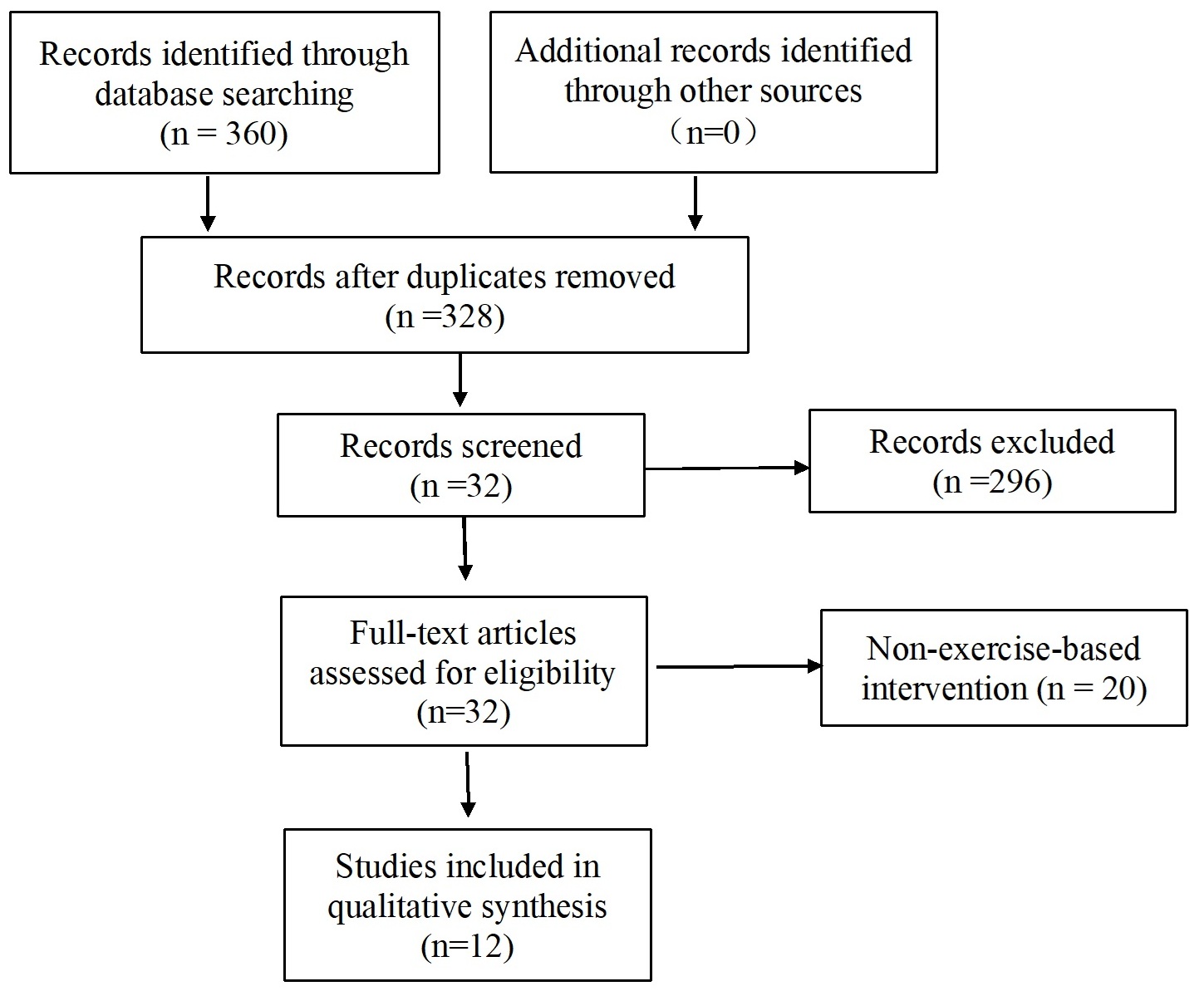

Supplement: Supplementary file 2 [file Image_1.jpeg]
